# Suppression of Paclitaxel-Induced Neuropathy and Ovarian Tumor Growth by Mn Porphyrin, MnTnBuOE-2-PyP^5+^ (BMX-001)

**DOI:** 10.1155/omcl/6333148

**Published:** 2025-08-24

**Authors:** Ivan Spasojevic, Zhiqing Huang, Welida Tamires Alves da Silva, Weina Duan, Li Du, Cathleen Chen, Jie Cao, Shasha Zhang, Hannah Lee, Gaomong Lo, Artak Tovmasyan, Huaxin Sheng, Ines Batinic-Haberle, Angeles Alvarez Secord

**Affiliations:** ^1^Department of Medicine–Division of Oncology and Duke Cancer Institute PK/PD Core Laboratory, Duke University School of Medicine, Durham 27710, North Carolina, USA; ^2^Division of Reproductive Sciences, Department of Obstetrics and Gynecology, Duke Cancer Institute, Duke University School of Medicine, Durham 27710, North Carolina, USA; ^3^Department of Anesthesiology, Multidisciplinary Neuroprotection Laboratories, Center for Perioperative Organ Protection, Duke University School of Medicine, Durham 27710, North Carolina, USA; ^4^Department of Translational Neuroscience, Barrow Neurological Institute, Phoenix 85013, Arizona, USA; ^5^Department of Radiation Oncology, Duke University School of Medicine, Durham 27710, North Carolina, USA; ^6^Division of Gynecologic Oncology, Department of Obstetrics and Gynecology, Duke Cancer Institute, Duke University School of Medicine, Durham 27710, North Carolina, USA

**Keywords:** BMX-001, cellular and mouse tumor studies, Mn porphyrin, MnTnBuOE-2-PyP^5+^, mouse neuropathy model, ovarian high-grade serous CAOV2 cancer, SOD mimic

## Abstract

Numerous cellular and animal studies demonstrated the ability of redox-active Mn(III) *N*-alkyl- and *N*-alkoxyalkylpyridyporphyrins (MnPs) to protect normal tissue while suppressing tumor growth. The mechanism primarily involves the modulation of NF-кB and Nrf2 signaling pathways via catalysis of MnP/H_2_O_2_-driven protein thiol oxidation. Such differential protection/suppression effects have paved the way of Mn porphyrins (commonly known as mimics of superoxide dismutase) into clinical trials, therefore introducing new line of therapeutics that are affecting cellular redox status/oxidative stress, rather than specific proteins. The most clinically advanced Mn porphyrin, Mn(III) *meso*-tetrakis(*N*-n-butoxyethyl-2-pyridyl) porphyrin (MnTnBuOE-2-PyP^5+^, BMX-001) has progressed into five Phase II clinical trials, two of those related to the injuries of central nervous system. Currently, no efficient treatment for chemotherapy-induced neuropathy is available in clinics. We therefore employed BMX-001 to assess its effect on paclitaxel (PTX)-induced neuropathy. Mechanical (Von-Frey filaments) and thermal (hot plate) stimulation, toxicity (body weight), muscular coordination and general physical condition (rotarod) of female CD-1 mice were evaluated over 3 weeks with 2 mg/kg daily dosing and also at clinically relevant dosing of 0.8 mg/kg given subcutaneously (SC) twice weekly after 1.6 mg/kg loading dose. Data revealed a significant ability of BMX-001 to suppress peripheral neuropathy and neuroinflammation. Importantly, while protecting peripheral tissue, BMX-001 suppressed the tumor growth of CAOV2 high-grade serous ovarian cancer in a mouse subcutaneous xenograft model. Previously, the strong anticancer effect was only seen when Mn porphyrins were combined with radiation, chemotherapy, and ascorbate (Asc). Our data further demonstrate that high-grade serous ovarian cancer is the first in vivo cancer thus far studied where redox-active Mn porphyrin, as a single agent, exhibits strong anticancer effect, comparable to that of PTX. The effect is presumably due to high tumor levels of BMX-001 and high oxidative stress specific to the aggressive chemoresistant CAOV2 cell line. Such a strong anticancer effect of BMX-001 would allow for lowering the dosing of PTX and reducing the neuropathy. The combined neuropathy protection and anticancer efficacy demonstrate, therefore, strong therapeutic potential of BMX-001 for gynecological cancers. Moreover, the ability of BMX-001 to suppress neuropathy may be relevant for all types of cancer where chemotherapeutics that induce neuropathy are used as a standard-of-care.

## 1. Introduction

A common dose-limiting complication of cancer chemotherapy is neurotoxicity in central and peripheral nervous systems, second only to myelosuppression, which undermines the otherwise successful anticancer therapy, resulting in its rescheduling or even withdrawal [1, 2]. Peripheral neuropathy affects 30%–40% of patients undergoing chemotherapy [2]. 25% of patients have cognitive impairment. This chronic neuropathy is characterized by bilaterally symmetrical sensory symptoms (such as numbness, tingling, and pain) appearing in the feet, or in both the feet and hands and occurs with chemotherapeutics across drug classes, such as paclitaxel (PTX), carboplatin (CB), and proteasome-inhibitor, bortezomib, via distinctly different antitumor mechanisms (reviewed by Fumagalli et al. [3]). Chronic painful neuropathy negatively affects the quality of life, interferes with daily activities, and in severe cases leads to disability.

No preventive or curative therapies are clinically available that would offer protection to normal tissue during chemotherapy for cancer [3]. Several neuroprotective strategies for peripheral neuropathy, though of marginal impact, are already in clinical use and are summarized in [4]. Further, the comprehensive review summarized NIH-supported clinical trials on neuropathy that were conducted between January 1, 2011, and May 22, 2019 (listed on ClinicalTrials.gov database) [1]. The review revealed only one drug that modestly suppressed neuropathy, dual serotonin–norepinephrine reuptake inhibitor Duloxetine, which is recommended by clinical guidelines for the symptomatic treatment of painful chemotherapy-induced peripheral neuropathy [5]. Taxane-based drugs were most frequently studied preclinically, with majority of those investigating PTX [1]. No success was found in translating mechanistic information into the development of interventions [1]. Among common mechanisms studied were mitochondrial changes, glial cells, inflammation, and oxidative stress. Although the mechanism of action was stated in 10 of the clinical trials, only three trials identified a specific underlying mechanism of action (anti-inflammatory, oxidative stress) to justify the choice of intervention studied [1].

Wealth of data have demonstrated the protection of normal tissue by Mn porphyrins during radio- and chemotherapy [6, 7], while suppression of the tumor growth in animal models of glioma, head and neck, breast, anal and prostate cancers when combined with radiation, chemotherapy, and exogenous ascorbate (Asc) [6, 7]. The efficacy of Mn porphyrins in cellular studies on nonsmall cell lung cancer [8] and ovarian cancer [9] was also reported. Such differential effects reportedly arise from different levels of oxidative stress/H_2_O_2_ and Mn porphyrins in tumor versus normal tissue, which are utilized for Mn porphyrin/H_2_O_2_-driven catalysis of oxidation of cysteines of signaling proteins, such as NF-кB and Nrf2/Keap1. The oxidative modification of proteins modulates their activities, that is inhibits NF-кB while activating Nrf2 pathway [6, 7]. Such abundance of data supported the clinical development of two of the most efficacious Mn porphyrins that exhibited good safety/toxicity profiles: Mn(III) *meso*-tetrakis(*N*-n-butoxyethylpyridinium-2-yl) porphyrin, MnTnBuOE-2-PyP^5+^ (BMX-001), and Mn(III) *meso*-tetrakis(*N*-ethylpyridinium-2-yl) porphyrin, MnTE-2-PyP^5+^ (BMX-010). While MnTE-2-PyP^5+^ is in Phase II noncancer clinical trial (atopic dermatitis and itch, NCT03381625), MnTnBuOE-2-PyP^5+^ is in five Phase II trials on patients bearing high-grade glioma (HGG, Phase II, NCT02655601), head and neck (Phase I/II, NCT02990468), anal cancer (Phase I/II, NCT03386500), rectal cancer (Phase II/III, NCT05254327), and multiple brain metastases (Phase II, NCT03608020). The Phase II glioma trial, where BMX-001 was combined with radiation and temozolomide, was recently finalized with encouraging data that support the orphan, fast-track, and breakthrough status assigned to BMX-001 by the FDA [10]. Promising preclinical and clinical data on BMX-001, along with well-established action in reducing oxidative stress in cellular and animal models, were accompanied by the reduction in neuroinflammation of the normal tissue exposed to radiation or chemotherapy and have prompted us to explore its impact on PTX-induced peripheral neuropathy [7].

The studies by us and our colleagues have already indicated that BMX-001 may bear a therapeutic potential in gynecologic cancers [7, 11]. In a collaborative study, Luksana Chaiswing and her team reported that BMX-001, as a single drug, suppressed the growth of aggressive high-grade serous OV90 and OV90-derived, chemoresistant OVCD ovarian cancer cell lines [9, 12]. Importantly, under identical conditions, BMX-001 protected normal ovarian epithelial cell line, hTER7, against several chemotherapeutics, including CB and PTX. The surprisingly strong anticancer effect of BMX-001 as a single drug, which has not been previously seen with other cancers [6, 7], was due to high H_2_O_2_ levels demonstrated in aggressive OV90 and OVCD cell lines [9]. High tumor levels of H_2_O_2_ are critical for Mn porphyrin-driven protein cysteine oxidation with subsequent suppression of tumor growth [6, 7]. In other words, additional sources of oxidative stress, such as radiation, chemotherapy, or exogenous Asc, may not be required for Mn porphyrin-driven ovarian tumor growth suppression. With lower dosing of standard-of-care PTX therapy, the damage imposed on normal tissue could be significantly diminished, while further reduced by protective action of Mn porphyrin. The protection of normal tissue (adjacent to tumor) by Mn porphyrins has been previously reported when different tumors were exposed to radiation or chemotherapy. Such protection was herein substantiated on suppression of chemotherapy-induced damage to peripheral nerve tissue by Mn porphyrin, BMX-001 [6, 7].

Mechanistic studies conducted in collaboration with Margaret Tome's team on lymphoma were critical for understanding the differential impact of MnP in cancer vs. normal tissue [6, 7]. The work demonstrated that MnTE-2-PyP^5+^ enhanced glucocorticoid-induced apoptotic processes of lymphoma cells by employing H_2_O_2_ and glutathione to catalyze the oxidation/*S*-glutathionylation of cysteines of NF-кB (nuclear factor kappa B) with its subsequent inactivation. Such an effect was not seen in normal lymphocytes [6, 7]. Tome's team also showed that MnTE-2-PyP^5+^/H_2_O_2_-driven oxidation/S-glutathionylation of mitochondrial complexes I and III inhibited their activities; several other proteins were oxidized as found by proteomics analysis [6, 7]. The higher the levels of H_2_O_2_ and Mn porphyrin, the higher the extent of protein oxidation. H_2_O_2_ could be either endogenously produced or its levels could be increased via radiation, chemotherapy, or exogenously added Asc [13]. Extensive studies were subsequently done to demonstrate the extent of protein oxidation in a 4T1 mouse breast cancer cell line exposed to MnTE-2-PyP^5+^/Asc system, cycling of which gave rise to H_2_O_2_ [6, 7]. Briefly, the 3605 peptidyl cysteines were affected, out of which 1577 were oxidized 1.3-fold or higher [6, 7]. The cysteines (C) of transcription factors NF-кB (C59) and Nrf2-Keap1 (nuclear factor erythroid 2-related factor 2/kelch-like ECH-associated protein 1) (C288 of Keap1) were oxidized. Subsequent study corroborated such data on proteins and glutathione oxidation associated with suppression of 4T1 cell viability and tumor growth by MnTE-2-PyP^5+^/Asc/radiation and BMX-001/Asc/radiation [13].

The goal of this work was to (1) demonstrate that BMX-001 protects normal tissue, that is, the peripheral nerve system, thereby inhibiting PTX-induced peripheral neuropathy at clinically relevant dosing, (2) provide evidence that BMX-001 does not protect ovarian tumor, and (3) address mechanism behind such effects. We used herein three sources of exogenous H_2_O_2_ in cellular and mouse cancer studies: Asc, Mn porphyrin (BMX-001), and chemotherapeutic, PTX. In a cellular study, we explored the aggressive high-grade (CAOV2) and low-grade serous ovarian epithelial (HOC7) cancer cell lines. Most of the epithelial ovarian cancers, which represent 85% to 90% of all ovarian cancers, are aggressive, high-grade serous cancers, with worse clinical outcome and primary and secondary chemoresistance, while 5%–10% of all serous ovarian cancers are of low-grade [14]. We have therefore used the high-grade serous CAOV2 cancer cell line to explore the impact of BMX-001/PTX/Asc on the tumor growth in a mouse subcutaneous xenograft model.

The reasoning behind using exogenous Asc in vitro and in vivo ovarian cancer studies is as follows. We and others have shown that Asc, when cycling with endogenous metalloporphyrins [15, 16], or with exogenously administered Mn porphyrins [6, 7] or when added along with chemotherapy/radiation [13, 17–20], gave rise to large amounts of H_2_O_2_, whereby increasing tumor oxidative stress and consequently suppressing its growth via Mn porphyrin/H_2_O_2_-driven catalysis of protein oxidation [21]. Relevant to ovarian cancer, Ma et al. [22] found, when Asc and CB were combined, the increased death of OVCAR5, OVCAR8, and SHIN3 ovarian cancer cell lines compared to either drug alone. In an athymic mouse model of SHIN3 cell line, both drugs combined were again more efficacious than either alone [22]. Nauman G et al. [15] provided a review on the toxicity and effectiveness of the use of Asc alone and combined with chemotherapy in different cancers. A total of 23 trials involving 385 patients were described. Overall, Asc was safe alone and in combination with chemotherapy and efficacy data were promising. Yet, the single-arm trials were done, except for a trial where 27 patients bearing stage III or IV ovarian cancer were randomized to CB/PTX or CB/PTX/Asc. The patients were treated with Asc (75–100 g intravenously twice weekly for 12 months) concurrent with CB and PTX. The 8.75-month increase in progression-free survival and a trend in overall survival in Asc arm were observed; also, the chemotherapy-related toxicity was reduced by Asc [15, 22]. Two additional trials, which justified further research on Asc, were reported in different cancers, including ovary. The Phase I single-arm trial was done with Asc as a single drug [23], while Phase I/II single-arm trial was done with Asc combined with chemotherapy [24]; both trials are summarized in Nauman et al. [15]. The randomized Phase I/II clinical trial (with Asc/chemotherapy and placebo) is ongoing on breast cancer patients where intravenous Asc was supplemented to conventional neoadjuvant chemotherapy [15].

## 2. Materials and Methods

A high-grade serous CAOV2 and a low-grade HOC7 serous ovarian cancer line were obtained from the collection maintained by the Department of Obstetrics and Gynecology at Duke University. Both cell lines were genetically authenticated by DNA Sequencing and Analyses Core at the University of Colorado Denver (Denver, CO) in 2008. Genomic DNA isolated from the individual cell lines was amplified at short tandem repeats using the Applied Biosystems Identifier Kit and an ABI 3730 capillary sequencer. Freezer stocks were prepared from the authenticated cells and were subsequently used for the experiments described herein. Cells were also mycoplasma tested using the Lonza MycoAlert PLUS assay by the Duke Cell Culture Facility. Ovarian cancer cell lines were typically used no more than five passages after thawing and were grown in RPMI1640 medium with L-glutamine (Sigma–Aldrich) supplemented with 10% FBS in a humidified incubator with 5% CO_2_ at 37 °C. The HOC7 cell line is of low-grade origin. Yet, it bears KRAS (Ki-ras2 Kirsten rat sarcoma viral oncogene homolog) and TP53 (tumor suppressor protein 53) mutations, the latter being characteristic of high-grade cell lines. It is thus known as putative low-grade cell line [14, 25]. Sodium L-Asc (>98%) was obtained from Sigma–Aldrich. CB (J code #J9045, aqueous solution) and PTX (NDC 45063-613-59, dissolved in anhydrous ethanol at 6 mg/mL) were obtained from Drugs.com through DLAR pharmacy at Duke University. CB and PTX were then diluted to appropriate concentrations in culture medium or saline, respectively. The GMP-grade BMX-001 (good manufacturing practice) was used.

Duke University's Division of Laboratory Animal Resources (DLAR), where animals have been housed with professional veterinary support, has a continuously accredited program from AAALAC International. All experiments using animals were performed according to the approved IACUC protocol for humane care and use of animals. For humane care and use of animals, the IACUC protocol #A131-20-06 for neuropathy mouse studies, and the IACUC protocol #A054-17-03 for mouse cancer studies were approved. In addition to the assessment of neuropathy and cancer growth, mice's overall health (as specified by Duke Department of Laboratory Animal Research, DLAR) was observed daily, and their weight and muscular coordination (via rotarod) were measured weekly as stated in detail under relevant sections of Section 2. The manuscript was written according to ARRIVE reporting guidelines, https://arriveguidelines.org/sites/arrive/files/documents/Author%20Checklist%20-%20Full.pdf.

### 2.1. Peripheral Neuropathy

#### 2.1.1. Mouse Study

The peripheral neuropathy was evaluated using standard methods via thermal (hot plate) [26] and mechanical stimulus (Von-Frey filaments) [27, 28]. Briefly, the hot plate was preheated to 52 °C. Mice were placed on the top surface of the hot plate, and the timer started. When mice felt discomfort, they raised the paw and licked it. The latency from the paw placement to the paw withdrawal was recorded; the measurement was done in triplicate. Von-Frey measurements were performed by “up-and-down” method using filaments of different thickness (Stoelting, ranging from 3.61 [0.407 g] to 5.46 [26 g] bending force). The test was performed on front and hind paws in duplicates. The toxicity (body weight), muscular coordination, and general physical condition (rotarod) measurements were assessed before treatment, and then weekly until the study was completed. The rotarod measurements were done in triplicate.

To eliminate drug formulation (vehicle) effects, all animal groups were exposed to both subcutaneous (SC) and intraperitoneal (IP) injections of vehicles for BMX-001 and PTX, respectively, with or without drugs. The standard U.S.P. vehicle for IP mouse PTX injections was prepared as an appropriate mixture of kolliphor/ethanol/citric acid in saline. The standard vehicle for BMX-001 was saline given SC. In each trial, 10 animals per group were used. Before treatment, animals were randomized to groups based on average body weight.

Initially, a pilot study was performed to identify whether the anticipated signal dynamic range would be sufficient to allow for measurements of the neuroprotective effect of BMX-001. The study was performed with two groups of 6-week-old female CD-1 mice (10 mice per group) obtained from Charles River. The mice were treated with either vehicle or 10 mg/kg PTX. The treatment was given IP, four times every other day, and body weight and neuropathy measurements were performed before treatment and at weeks 1, 2, 3, and 4. The study produced statistically significant effect (signal “gap”) when compared to vehicle-only group as measured by both Von-Frey measurements (mechanical allodynia) and hot plate (thermal allodynia) tests. The excellent agreement between Von-Frey and hot plate results is important because the two tests assess allodynia in different ways, that is, by mechanical versus thermal stimulation, thus validating the overall result. Neither the significant toxicity (body weight loss) nor the drop in rotarod performance was observed at this PTX dosing regimen (data not shown). A third-week experiment was accepted as appropriate to develop sufficient allodynia effect, as the fourth week did not provide any additional information.

Three trials were subsequently performed. In the first two trials, BMX-001 was given SC at 2 mg/kg/day 5 days per week over the period of 3 weeks. In a third trial, a dosing regimen more relevant to clinical trials was adopted. Namely, from available human Phase I plasma PK data, we were able to estimate the dose that would provide equivalent BMX-001 exposure (AUC) in mouse. By obtaining AUC_human_ (0.2 mg/kg)/AUC_mouse_ (0.2 mg/kg) = 4.5 and AUC_human_ (0.4 mg/kg) /AUC_mouse_ (1 mg/kg) = 3.3 (unpublished data), we accepted factor 4 as average human-to-mouse dose adjusting factor. Thus, the established maximal tolerable subcutaneous dose (MTD) in humans, 0.4 mg/kg loading + 0.2 mg/kg twice weekly, translates to equivalent mouse subcutaneous dose: 1.6 mg/kg loading + 0.8 mg/kg twice weekly.

In Trials 1 and 2, 2 mg/kg/day of BMX-001 was given SC five times per week (from Monday to Friday). The four groups (*n* = 10) of 6-week old female CD-1 mice from Charles River were treated as follows: (1) vehicle group, no drugs, receiving four IP injections of PTX-vehicle, every other day, and daily SC injections of vehicle for BMX-001 (saline); (2) PTX group, receiving four IP injections of 10 mg/kg PTX given every other day, and daily SC injections of saline; (3) BMX-001 group, receiving four IP injections of PTX-vehicle every second day and daily SC injections of 2 mg/kg/day BMX-001; and (4) BMX-001/PTX group, receiving four IP injections of 10 mg/kg PTX given every other day, and daily SC injections of BMX-001. At the end of the study, spinal cords and paws were collected.

In Trial 3, the 1.6 mg/kg loading dose of BMX-001 was given on the first Monday, and the dosing was continued at 0.8 mg/kg twice weekly on Mondays and Fridays for the continuation of the study. Thus, the total dose given in Trial 3 was 6.25-fold lower than in Trials 1 and 2. The four groups (*n* = 10) of 6-week old female CD-1 mice from Charles River were treated as follows: (1) vehicle group, no drugs, receiving four IP injections of PTX-vehicle, every other day, and daily SC injections of BMX-001-vehicle on first Monday, continued subsequently on Mondays and Fridays; (2) PTX group, receiving four IP injections of 10 mg/kg PTX given every other day, and SC injection of BMX-001-vehicle on first Monday, continued subsequently on Mondays and Fridays; (3) BMX-001 group, receiving four IP injections of PTX-vehicle every second day, and loading SC injection of 1.6 mg/kg BMX-001 on first Monday continued twice weekly on Mondays and Fridays at 0.8 mg/kg; and (4) BMX-001/PTX group, receiving four IP injections of 10 mg/kg PTX given every other day, and loading SC injection of 1.6 mg/kg BMX-001 on first Monday continued twice weekly on Mondays and Fridays at 0.8 mg/kg. At the end of the study, spinal cords and paws were collected.

All three trials were performed by the same operator who was blinded to the identity of cages and animals tested. Another person, not familiar with the study details, was responsible for generation of keys and scrambling of cage numbers and for entering the unblinded test records into the data system. In all three studies, a full “mouse conditioning/training trial” of Von-Frey, hot plate, and rotarod tests was performed 2 days before the week 0 tests. No anesthesia was used for neuropathy testing as no serious pain was inflicted during the tests, and because mild discomfort was part of the experiment and anesthesia would introduce a bias into the measurements. At the end of the studies, mice were euthanized by deep terminal anesthesia using 5% isoflurane with cervical dislocation as a secondary euthanasia method. Simple IP and SC bolus injections did not require anesthesia, according to the approved animal protocol. Dead animals were disposed of by Duke University's Division of Laboratory Animal Resources. Statistical analysis was performed on GraphPad Prism v.10. Two-way ANOVA and Tukey's multiple comparisons were performed for analysis of data between the four study groups on weeks 0–3. Multiple measurements, *n* = 4 in case of Von-Frey test, and *n* = 3 for other tests, were averaged so a single value for the mouse (*n* = 10)/week was used in ANOVA calculations. For PTX and PTX/BMX-001 groups on weeks 2 and 3, normality (within GraphPad) and homogeneity of variance (Levene's test) was also performed. For a full statistical report, please refer to Table S1.

**Scheme 1:**
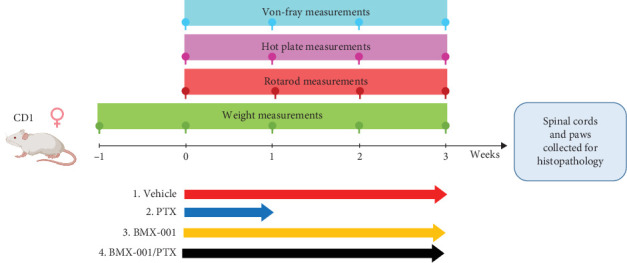
Timeline of the treatment of mice with BMX-001 and paclitaxel (PTX) in a neuropathy study. In Trials 1 and 2, 2 mg/kg/day BMX-001 was given via SC injection 5 days per week. In Trial 3, BMX-001 was given SC as 1.6 mg/kg loading dose on first Monday, and the 0.8 mg/kg dosing was continued twice weekly on Mondays and Fridays. The treatment details are provided under materials and methods in Section 2.1.1.

Scheme 1 depicts the timeline of the treatment of mice with BMX-001 and PTX, in a neuropathy study.

#### 2.1.2. Histopathology

At the end of neuropathy Trial 2, histopathology was assessed in the dorsal horn of spinal cords as reported [26, 27]. Tissue was taken from five CD-1 mice under deep terminal isoflurane anesthesia with 5% isoflurane, followed by whole body perfusion with saline and excision of tissues. A series of 30 µm sections was cut from the tissue using cryostat and stored in PBS. Before immunohistochemical staining, the sections were immersed in 10% donkey serum (EMD Millipore, USA) for 30 min to block nonspecific binding. Then, they were transferred to the primary antibody solution at 4 °C overnight. The cytoplasmic ionized calcium-binding adaptor molecule 1 (Iba-1, 1:500, Fujifilm Wako Chemicals, USA) was used to measure microglia. Astrocytes were assessed by immunofluorescence with monoclonal glial fibrillary acidic protein, GFAP, antibodies (1:500, Agilent Technologies Denmark). The member of the ubiquitin hydrolase family of proteins, protein gene product PGP 9.5, was used for immunostaining of paw skin nerve fibers (PGP 9.5 polyclonal antibody, 1:500, Invitrogen). Immunohistochemistry was also used to measure the proinflammatory cytokines: tumor necrosis factor-α (TNF-α; 10 µg/mL) and interleukin-1β (IL-1β; 10 µg/mL, R and D Systems, Bio-Techne, USA). We were not able to detect IL-1β signal in tissues from mice. TUNEL assay was performed to evaluate the extent of apoptotic death according to the manufacturer's protocol (TUNEL Assay kit, Fluorescence, 488 nm, Cell Signaling). On the second day, the sections were washed in PBS three times for 10 min and then transferred to the second antibodies (1:500, Alexa Fluor 488 donkey antigoat IgG or Alexa Fluor 594 donkey antirabbit IgG) for 2 h at room temperature. After three times of PBS washing, the sections were mounted on the slides, and a drop of fluoroshield with DAPI (Sigma, USA) was added before cover glass placement. One-way ANOVA was used for statistical analysis.

### 2.2. Cancer Suppression-In Vitro Cellular Studies

#### 2.2.1. Viability of CAOV2 and HOC7 Ovarian Cancer Cell Lines

The high-grade serous CAOV2 and low-grade serous HOC7 ovarian cancer cell lines were seeded onto 96-well plate with RPMI 1640 medium plus 10% fetal bovine serum and 1% penicillin and streptomycin. The chemotherapeutic drugs were added to the cells after 24 h of cells plating and the treatment was applied for 72 h. The nontoxic concentrations of single drugs (BMX-001, PTX, CB, and Asc) were used to demonstrate whether there would be a benefit (synergism) of double and triple combinations. Two tests were done at different concentrations of BMX-001, PTX, CB, and Asc. The concentrations of BMX-001, PTX, CB, and Asc used for Dosing 1 and Dosing 2 (in parenthesis) were as follows: BMX-001 = 2.5 (5) µM, Asc = 0.25 (0.5) mM, PTX = 50 (100) nM, and CB = 7.5 (15) µM. The cell viability was assessed using CellTiter-Glo Luminescent Cell Viability Assay (Promega) according to the protocol provided by the manufacturer. The relative cell viability was calculated against no-treatment control. The average values at each dose were calculated from 64 replicates and normalized against control values. Statistics. The paired Student's *t*-test was performed. Double treatments and triple treatments were compared to each other and to single treatments with BMX-001.

#### 2.2.2. Expression of NF-кB, Nrf2, IL-1β, and BLC2 Proteins

The expression of NF-кB, Nrf2, and BCL2 was determined by Western Blotting when CAOV2 ovarian cancer cells were treated with either 5 µM BMX-001 or 100 nM PTX or their combination for 72 h. The cell lysate was prepared after cell treatment, and the proteins were separated by gel electrophoresis using 4%–15% precast polyacrylamide gel (Bio-Rad, cat#4561086) and 100 µg of cell lysate. Western blotting with specific antibodies against NF-кB, BCL2, and Nrf2 was performed according to the protocols (https://www.abcam.com/protocols/general-western-blot-protocol), and GAPDH was used as the loading control. After overnight transfer to nitrocellulose membranes, the blots were incubated with primary antibodies, including anti-NF-кB 1:1000 (Abcam, Cat# ab16502), anti-BCL2 1:1000 (Abcam, Cat# ab196495), anti-Nrf2 1:100 (Santa Crus, sc-365949), IL-1β polyclonal antibody 1:500 (ETA 3-24-23 and anti-GAPDH 1:2500 (Abcam, Cat# ab9485) for 2 h at room temperature. After the incubation with secondary antibody, ECL Western Blot Substrate kit (Bio-Rad, Cat# 1705060S) was used to detect the signals, which were imaged using the Chemidoc imaging system from Bio-Rad.

### 2.3. Cancer Suppression-In Vivo Mouse Studies

#### 2.3.1. Suppression of Tumor Growth

The athymic nude female mice, 4–6 weeks of age were obtained from Charles River. The 5 × 10^6^ CAOV2 ovarian tumor cells in 100 µL were implanted into mice flanks under mild anesthesia using 2% isoflurane via nose cone. Treatments started when tumors reached on an average of 40 mm^3^. Animals were randomized to groups based on average tumor size. For sufficient statistical power, 20 mice per group were used, which, for logistical reasons, limited the number of groups to four per study. Two studies were performed.

**Scheme 2:**
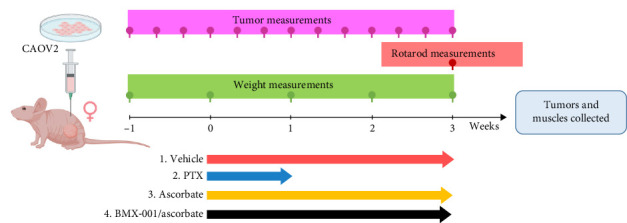
Timeline of the mouse xenograft CAOV2 ovarian tumor growth suppression with BMX-001 ascorbate (Study 1). The treatment details are given under materials and methods in Section 2.3.1.

Study 1 was designed to evaluate the impact of PTX, Asc, and Asc combined with BMX-001 (Scheme 2). Four groups of 6-week old female mice were treated as follows: (1) vehicle group received daily PTX/Asc-vehicle IP saline injections, and daily BMX-001-vehicle SC injections of saline; (2) PTX group received IP injections of 20 mg/kg PTX given every other day for 5 days and continued with daily IP and SC injections of saline; (3) Asc group received IP injections of 1 g/kg/day of Asc and SC injections of saline; and (4) BMX-Asc group, received 1 g/kg/day IP injections of Asc and SC injections of 2 mg/kg/day BMX-001. Treatments started when tumors reached an average volume of 40 mm^3^. The 2 mg/kg/day dosing of BMX-001 is the one used in neuropathy studies and previously in our mouse studies of 4T1 breast cancer and glioma [6, 7, 13].

**Scheme 3:**
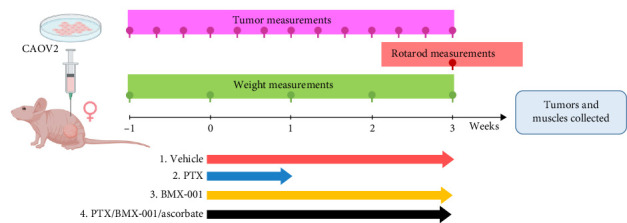
Timeline of the treatments in the mouse ovarian subcutaneous xenograft CAOV2 tumor growth study (Study 2). Mice were treated with BMX-001, paclitaxel and ascorbate as described under materials and methods in Section 2.3.1.

In Study 2, PTX and BMX-001 were used as single drugs and were then both combined with Asc to further increase the tumor oxidative stress (Scheme 3). The groups of mice were as follows: (1) vehicle mice were treated with IP saline injections (vehicle for PTX and Asc) and SC saline injections every day (vehicle for BMX-001); (2) PTX group of mice received five IP injections of 20 mg/kg/day PTX given every second day for 5 days, continued with IP and SC saline injections every day; (3) BMX-001 group of mice were treated with SC injections of 2 mg/kg/day BMX-001 and IP saline every day; and (4) PTX/BMX-001/Asc group of mice received five IP injections of 20 mg/kg PTX given every second day, SC injections of 2 mg/kg/day BMX-001 and IP injections of Asc at 1 g/kg/day. Treatments started when tumors reached an average volume of 130 mm^3^. Tumor volumes and mouse weights were measured every second day. At the end of the study, on day 22, the well-being of mice was measured on rotarod. On day 22, the tumors and muscles from the leg opposite to the tumor-bearing leg were excised and snap-frozen for determination of the BMX-001 levels. At the end of cancer studies, mice were euthanized with 59 µL of euthasol solution per mouse, which contains at least 250 mg/kg pentobarbital, via IP route with cervical dislocation as a secondary method, per approved animal protocol. Dead animals were disposed of by Duke University's Division of Laboratory Animal Resources. The one-way ANOVA test within GraphPad Prism v.10 software was used for statistical analysis, and the data expressed as mean ± SEM, *⁣*^*∗*^*p* < 0.05; *⁣*^*∗∗*^*p* < 0.01.

#### 2.3.2. BMX-001 Levels in Tumor and Nontumor Tissues

LC-MS-MS was used for determination of the levels of BMX-001 in tumors and muscles from nontumor-bearing leg. The tissues were excised and snap-frozen at the end of Study 2, where the effect of PTX, BMX-001, and both combined with Asc was studied. Ten mice were analyzed for BMX-001 levels in muscle and tumor of mice treated with BMX-001 or with BMX-001/PTX/Asc. The method is described in detail in [13].

## 3. Results

### 3.1. Peripheral Neuropathy

#### 3.1.1. Suppression of PTX-Induced Peripheral Neuropathy

**Figure 1:**
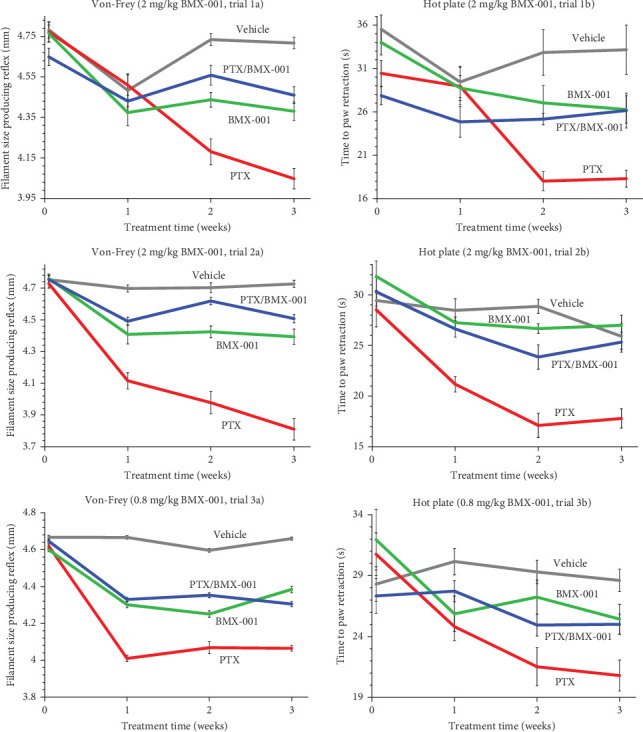
The efficacy of BMX-001 in reducing PTX-induced neuropathy. The impact of BMX-001 on PTX-induced neuropathy was measured with hot plate (thermal allodynia) and von Frey filaments (mechanical allodynia). The toxicity was assessed by body weight, while muscular coordination and general physical condition was evaluated by rotarod (Figure S1). Ten mice per group were studied. The BMX-001 was given SC at 2 mg/kg/day in Trials 1a/1b and 2a/2b. In Trial 3a/3b, the loading dose of 1.6 mg/kg of BMX-001 was given on a first Monday and was followed by twice weekly SC dosing at 0.8 mg/kg on Fridays and Mondays. In all trials, the PTX was given IP at 10 mg/kg every second day for four days. All measurements were made before the treatments (Week 0) and then once a week for 3 weeks afterwards. All data plotted are presented as mean ± SEM. Two-way ANOVA was performed on GraphPad Prism v.10. Tukey's multiple comparisons tests were performed to determine differences in multigroups experiments; for a full report please refer to Table S1.

Trials 1 and 2 were done at 2 mg/kg/day of subcutaneous dosing of BMX-001 (Figure 1). Such dosing has been commonly used in all mouse studies where protection of normal tissue and anticancer effect of BMX-001 were explored. Rotarod performance and body weights for Trials 1 (1c and 1d) and 2 (2c and 2d) are detailed in Figure S1 [6, 7, 13]. The subcutaneous route of administration is identical to the one used in clinics, yet the dosing was larger. To demonstrate the clinically relevant therapeutic potential of BMX-001 in reducing chemotherapy-induced neuropathy, we performed Trial 3 with loading dose of 1. 6 mg/kg followed by 0.8 mg/kg, given twice per week. In this way, we reduced cumulative dosing 6.25-fold relative to 2 mg/kg/day studies (Figure 1, Trials 3a and 3b, and Figure S1, Trials 3c and 3d). For a full report on statistical analysis of neuropathy study, please refer to Table S1.

In all three trials, (1) 2 mg/kg BMX-001 daily, (2) 2 mg/kg BMX-001 daily (repeated), and (3) 0.8 mg/kg BMX-001 twice a week, mechanical allodynia (Von-Frey test) was significantly improved in PTX/BMX-001 vs PTX-only group (Figure 1 and Table 1). Thermal allodynia testing (hot plate) resulted in the same conclusion, except in case of trial 3 (0.8 mg/kg BMX-001), where *p* < 0.05 criterion was not reached (Table 1). Nevertheless, the trend (Figure 1, plot 3b) strongly suggests that the statistical power and logistical limitations, but not the lack of effect, are responsible. Importantly, there was no significant difference in rotarod performance and body weight between experimental groups over the entire experiment, attesting to the absence of systemic toxicity, which would have otherwise introduced bias in the peripheral neuropathy assessment. Data on rotarod performance and body weight in all three trials and the full statistical analysis of all neuropathy testing are provided in Figure S1 and Table S1. The similarity of the effect at two dosing strengths is in support of Mn porphyrin acting as a catalyst, that is, not as a stoichiometric reactant. During the catalytic protective redox-cycling, after oxidation of protein thiols, BMX-001 gets re-oxidized primarily by oxygen [7].

#### 3.1.2. Histopathology

Mechanism of neuropathy, reviewed in detail by Fumagalli et al. [3], may involve mitochondrial damage, impairment of axonal transport, oxidative stress, and drug transporters. Recently, neuroinflammation has emerged to play a role in chemotherapy-induced neuropathy [3]. Glial cells appear to contribute to the maintenance of the neuroinflammatory processes in both the peripheral nervous system and spinal cord [3, 29, 30].

PTX is known to activate NF-кB via increased phosphorylation of NF-кB (p65 subunit), thereby upregulating proinflammatory cytokines such as TNF-α, IL-1β, and IL-6 [11, 31, 32]. Those cytokines were reportedly increased in PTX-induced neuropathy but are subsequently downregulated by MnTE-2-PyP^5+^ [11].

**Figure 2:**
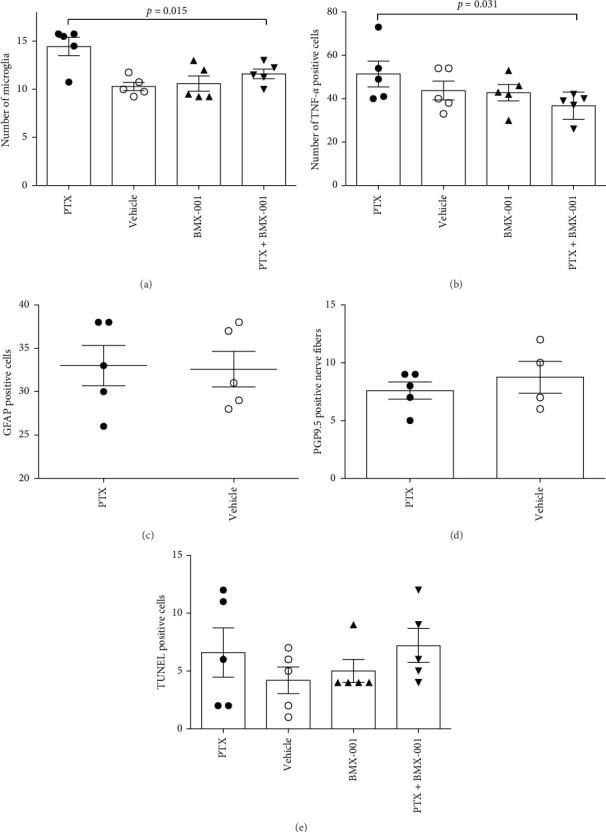
BMX-001-driven suppression of neuroinflammation induced by paclitaxel. BMX-001 reduced microglia (A) and TNF-α levels (B) in dorsal horn of spinal cord. The effect of PTX on astrocytes (C, GFAP positive cell), paw nerve fibers (D, PGP9.5 marker) or apoptosis (E, Tunel assay) was not observed. Spinal cords and paw skins were excised at the end of a mouse study on PTX-induced neuropathy. The study was done under conditions depicted in Figure 1 (Trial 2), and five mice were analyzed. One-way ANOVA was used for statistics.

In trial 2, we evaluated the impact of PTX, in the presence and absence of BMX-001, on neuroinflammation in the dorsal horn of the spinal cord (the location of the sensory synapses) and in paw skin. Our data (Figure 2) demonstrated that PTX increased neuroinflammation of spinal cord dorsal horn via activation of microglia and increase in key proinflammatory cytokine, TNF-α. The BMX-001, however, reduced PTX-induced activation of microglia and the levels of TNF-α. No impact of PTX on astrocytes (GFAP), apoptosis (TUNEL assay), and paw skin nerve fibers (PGP 9.5) was found (Figure 2).

### 3.2. Cancer Suppression

#### 3.2.1. Cellular Studies

##### 3.2.1.1. Inhibition of the Viability of High-Grade CAOV2 and Low-Grade HOC7 Serous Ovarian Cancer Cell Lines When Exposed to Sources of H_2_O_2_

**Figure 3:**
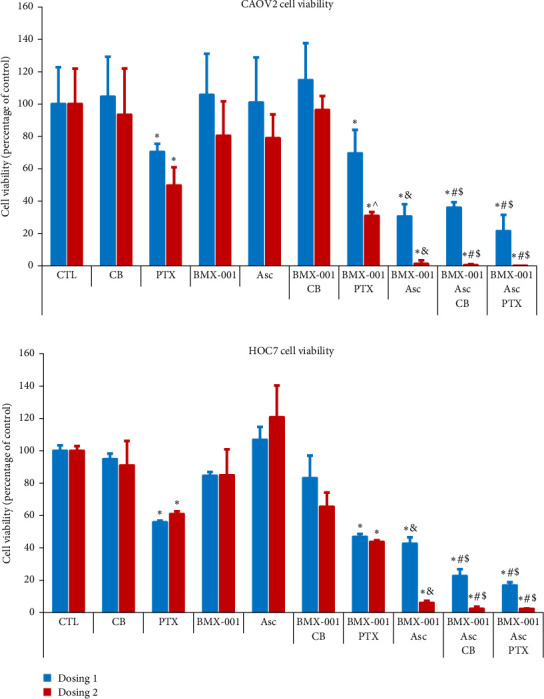
MnTnBuOE-2-PyP^5+^, combined with sources of H_2_O_2_ (ascorbate and/or paclitaxel), suppresses growth of high-grade CAOV2 and low-grade HOC7 serous ovarian cancer cell lines. Ovarian cancer cells were plated for 24 h and treated for 72 h. Abbreviations: BMX-001 (MnTnBuOE-2-PyP^5+^), PTX (paclitaxel), CB (carboplatin), Asc (ascorbate), CTL (control). Concentrations for Dosing 1 and Dosing 2 (in parenthesis) for COAV2 cells are: BMX-001 = 2.5 (5) µM, Asc = 0.25 (0.5) mM, PTX = 50 (100) nM, CB = 7.5 (15) µM, and for HOC7 cells are: BMX-001 = 5 (10) µM, Asc = 0.25 (0.5) mM, PTX = 250 (500) nM and CB = 50 (100) µM. Statistics. Study was done in four replicates and the *p* values were shown as: *⁣*^*∗*^*p* < 0.05 for BMX-001/PTX (Dosing 1 and 2) versus control; PTX (Dosing 1 and 2) versus control; *⁣*^∧^*p* < 0.05 BMX/PTX (Dosing 2) versus PTX (Dosing 2) for CAOV2 cells; *⁣*^&^*p* < 0.05 versus BMX-001 or Asc treatments alone, *⁣*^#^*p* < 0.05 versus chemo (CB or PTX) and ^$^*p* < 0.05 versus BMX-001/Chemo (CB or PTX).

The study was done under concentration conditions where none or minimal impact of single treatments was seen, which allowed us to observe whether the synergism, when two or three drugs were combined, exists. The lower and higher concentrations of drugs were tested, indicated as Dosing 1 and Dosing 2, respectively. In addition to PTX, we also tested CB as it is another first-line treatment of advanced ovarian cancer. As shown in Figure 3, PTX was toxic to CAOV2 cells at 50 and 100 nM, while BMX-001 was neither toxic at 2.5 µM nor at 5 µM. A synergistic effect was seen on CAOV2 cell viability suppression when 100 nM PTX was combined with 5 µM BMX-001 (Dosing 2, *p*=0.02135). BMX-001 did not increase the effect of CB in CAOV2 cells, but it did so slightly, yet insignificantly, in HOC7 cells. In both cell lines, Asc imposed a profound effect on cell viability when combined with BMX-001. The effect was only slightly enhanced when either PTX or CB was added to BMX-001/Asc. It is well known that cancer cells are under oxidative stress (with higher H_2_O_2_ levels), and more so the aggressive, chemoresistant cell lines, such as CAOV2 (Section 1 [9]). Cancer cells control high oxidative stress with endogenous antioxidative defenses but are very sensitive to any additional exogenous sources of H_2_O_2_, which could promote apoptotic process [33]. Therefore, cancers are frequently treated with radio- and/or chemotherapy-based exogenous sources of H_2_O_2_. In our previous studies on aggressive triple negative 4T1 cell line [6, 7] as well as in this study, exogenous Asc was used as an additional source of H_2_O_2_. BMX-001 redox cycled with Asc thereby producing large amounts of H_2_O_2_ in a catalytic manner. Once overwhelming, the production of H_2_O_2_, via BMX-001-driven catalysis of protein oxidation, resulted in a suppression of tumor cell viability. As anticipated, based on the BMX-001/ovarian cancer cellular studies by Chaiswing et al. [9], the effect is more pronounced with high-grade serous and chemoresistant CAOV2 than with low-grade serous HOC7 ovarian cancer cell line. Importantly, Chaiswing et al. [9] reported that BMX-001 protected normal ovarian epithelial cells against PTX toxicity.

##### 3.2.1.2. Expression of NF-кB, Nrf2, and BLC2 Proteins

The study was performed to gain insight into the expression of major proteins, which were reportedly affected by Mn porphyrins in different cancers [7, 9]. Mn porphyrins inhibited NF-кB pathway in cellular studies of hematologic malignancies, breast cancer, and in mouse subcutaneous xenograft glioma study [6, 7]. Mn porphyrins further suppressed Nrf2 pathway in OV90 and OVCD ovarian cancer cell lines [9]. The oxidative modification of Nrf2-Keap1 pathway was also seen when 4T1 breast cancer cells were exposed to MnTE-2-PyP^5+^/Asc oxidizing system [7]. Finally, in a 4T1 breast cancer study and glioma mouse subcutaneous xenograft study, lipophilic MnP analogs suppressed levels of BCL2 [6, 7], which has been reportedly upregulated in ovarian cancer patients [34, 35].

**Figure 4:**
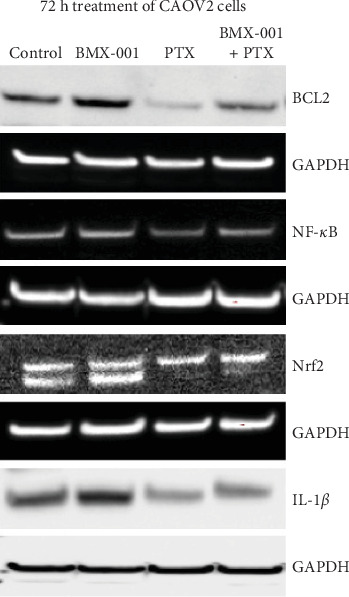
Expression of BCL2, NF-кB, Nrf2, and IL-1β as impacted by PTX, BMX-001, and PTX/BMX-001. The chemoresistant high-grade serous CAOV2 ovarian cancer cells were treated with either 5 µM BMX-001 or 100 nM PTX or both combined for 72 h. GAPDH was used as internal loading control.

The impact of PTX was explored when combined with BMX-001. The synergistic effect on suppression of cell viability was seen when 100 nM of PTX was combined with 5 µM BMX-001 in treatment of CAOV2 cells (Figure 3). We thus used these concentration conditions to assess the impact of PTX and BMX-001, as single drugs and combined, on expression of proteins. CAOV2 cells were exposed to PTX or BMX-001, or their combination for 72 h. Treatment with BMX-001 resulted in expression of prosurvival BCL2, NF-кB, Nrf2 proteins, and NF-кB-controlled IL-1β cytokine (Figure 4). PTX suppressed the expression of all four proteins relative to nontreated and BMX-001-treated cells. BMX-001/PTX treatment suppressed all four proteins, relative to nontreated and BMX-001-treated cells, but to a lesser degree than PTX (Figure 4).

We may safely assume, based on our previous studies, that proteins expressed in cells exposed to BMX-001/PTX (which system is the inducer of oxidative stress) were oxidized via BMX-001/H_2_O_2_-driven catalysis. Such oxidative modification reportedly modifies the activities of proteins. Specifically, MnTE-2-PyP^5+^ combined with another chemotherapeutic, glucocorticoid (dexamethasone), oxidized NF-кB in a S-glutathionylated manner [6, 7] and subsequently inhibited its activity. Further, the same system also induced S-glutathionylation with subsequent inhibition of mitochondrial proteins and depletion of cellular ATP in malignant human T-cells [6, 7]. Proteomics analysis of the same system demonstrated that many other proteins were also oxidized [6, 7]. Keap-1, NF-кB, and numerous proteins of 4T1 cells were oxidized by MnTE-2-PyP^5+^/Asc treatment [6, 7]. In a mouse 4T1 flank tumor study, MnTnHex-2-PyP^5+^/Asc/radiation downregulated BCL2 along with NF-кB and other prosurvival and metastatic proteins [6]. In another study, the suppression of BCL2, along with NF-кB, was also seen when 4T1 cells were treated with MnTnHex-2-PyP^5+^/radiation [6, 7]. The increase in total S-glutathionylated proteins was found in a 4T1 cellular and mouse cancer study with MnTE-2-PyP^5+^/Asc/radiation and MnTnBuOE-2-PyP^5+^/Asc/radiation [13]. The inhibition of Nrf2-Keap1 system was seen in the study of Chaiswing et al. [9] on two other aggressive ovarian cancer cell lines, OV90 and OVCD (as summarized in Section 1).

#### 3.2.2. Mouse Studies

##### 3.2.2.1. Suppression of Tumor Growth

Mice were treated with PTX, BMX-001, and Asc as single drugs and combined. Asc was tested because previous studies [6, 7, 13] demonstrated that, when combined with Mn porphyrins or Mn porphyrins/radiation, it contributed to the suppression of cellular viability. Those Mn porphyrins that cycle with Asc enhanced the radiation-mediated tumor growth suppression in cellular and mouse 4T1 breast cancer studies via oxidative damage to signaling proteins [6, 13]. The justification for studying Asc has also been based on the promising results from preclinical and ongoing clinical trials of Asc alone and combined with chemotherapy, as reviewed in Section 1.

**Figure 5:**
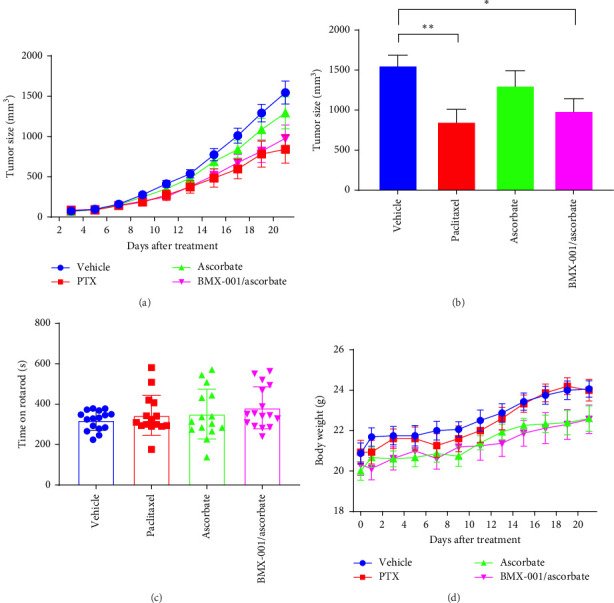
Ovarian tumor growth suppression with PTX and ascorbate as single drugs and BMX-001 combined with ascorbate (Study 1). (A) Tumor growth curves, (B) tumor size, (C) rotarod performance at the end of the study, and (D) mice weights. Tumor growth and weights are presented as Mean ± SEM. Four groups of mice (*n* = 20) were treated as follows: (1) vehicle group; (2) PTX group receiving 20 mg/kg PTX IP injections every other day for 5 days; (3) ascorbate group, receiving IP injections of 1 g/kg/day of ascorbate; and (4) BMX/ascorbate group, receiving 1 g/kg/day IP injections of ascorbate and SC injections of 2 mg/kg/day BMX-001. For treatment details see Section 2.3.1. One-way ANOVA paired test was used for statistics; *⁣*^*∗*^*p* < 0.05, *⁣*^*∗∗*^*p* < 0.01.

The growth of high-grade serous CAOV2 ovarian cancer cell line was followed in a mouse subcutaneous xenograft model under two conditions. In Study 1, the impact of Asc, PTX, and BMX-001 combined with Asc was explored. The cycling of BMX-001 with Asc aimed to increase tumor H_2_O_2_ levels, enhancing therefore BMX-001/H_2_O_2_-driven catalysis of oxidative damage to cellular proteins, promoting in turn apoptotic processes and tumor growth suppression (Figure 5). On day 21, Asc as a single drug did not significantly suppress tumor growth. Yet, PTX and MnTnBuOE-2-PyP^5+^/Asc significantly suppressed tumor growth by 48% (*p*=0.003) and 36% (*p*=0.014), respectively, when compared to vehicle group. No significant difference between groups was found with regard to mouse weights and their rotarod performance.

**Figure 6:**
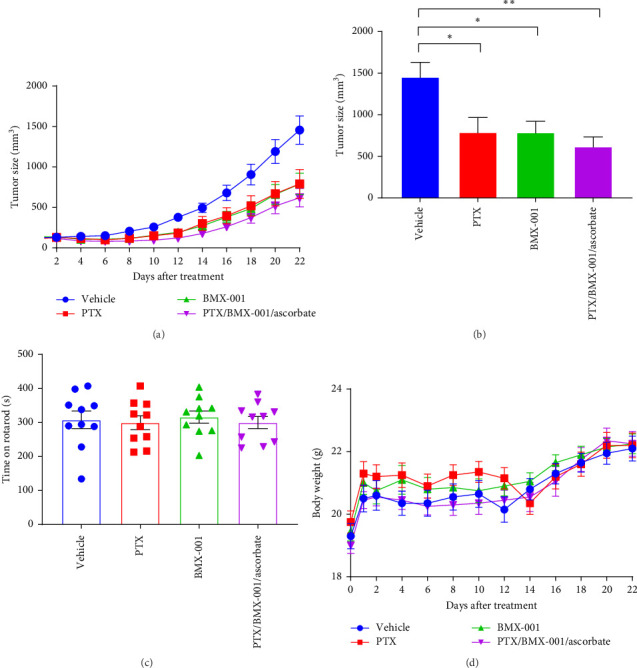
Ovarian cancer tumor growth suppression with BMX-001, PTX each as single drugs and both combined with ascorbate (Study 2). (A) Tumor growth curves, (B) tumor size, (C) rotarod performance at the end of the study, and (D) mice weights. Four groups (*n* = 20) of mice were as follows: (1) vehicle mice; (2) PTX group of mice received 20 mg/kg/day PTX given IP every second day for 5 days; (3) BMX-001 group of mice were treated with 2 mg/kg/day of BMX-001 given SC; and (4) PTX/BMX-001/ascorbate group of mice received IP injections of 20 mg/kg PTX every second day for 5 days, SC injections of 2 mg/kg/day BMX-001 and IP injections of ascorbate at 1 g/kg/day. For treatment details see Section 2.3.1. Tumor growth and weights are presented as mean ± SEM. One-way ANOVA paired test was used for statistics; *⁣*^*∗*^*p* < 0.05, *⁣*^*∗∗*^*p* < 0.01.

In Study 2, the effects of PTX, BMX-001, and PTX/BMX-001/Asc, the latter treatment being the triple source of H_2_O_2_, were compared (Figure 6). On day 22, tumor growth was significantly suppressed by PTX, BMX-001, and PTX/BMX-001/Asc. At that time, tumor volume was reduced by 46% in PTX-treated mice, 46% in BMX-001-treated mice, and 57% in PTX/BMX-001/Asc-treated mice (vs. vehicle mice) relative to vehicle group. Asc did not contribute significantly to tumor growth suppression by either BMX-001 or PTX under our experimental conditions. No significant difference was found between groups with regards to mice weights and their rotarod performance.

The robust anticancer effect of BMX-001 in suppressing tumor growth is presumably due to the high enough H_2_O_2_ levels in aggressive chemoresistant CAOV2 cell line that enabled the oxidative damage of cellular proteins, promoting tumor apoptosis. Such reasoning is based on a large effect of BMX-001, as a single drug, on suppression of cellular viability of aggressive ovarian cancer cell lines, OV90 and OV90-derived chemoresistant analog, OVCD. The effect was larger with OVCD, as it has higher H_2_O_2_ levels than OV90 [9].

The profound anticancer effect of BMX-001 might be of clinical relevance as it would allow for the lower PTX dosing in standard-of-care ovarian cancer therapy and would, in turn, reduce PTX-induced neuropathy.

##### 3.2.2.2. BMX-001 Levels in Tumor and Nontumor Tissues

**Figure 7:**
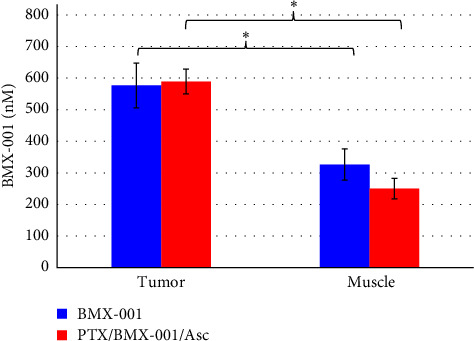
BMX-001 levels in tumor and muscle. Mice (*n* = 10) were treated as described in Figure 6. Tumors and muscles (from nontumor-bearing leg) were extracted at the end of the study. Statistics: (BMX-001 [muscle] versus BMX-001 [tumor], *p*=0.0096; PTX/BMX-001/Asc [muscle] versus PTX/BMX-001/Asc [tumor], *p*=0.000003) (see also further details on statistics in Table S2).

Both H_2_O_2_ and BMX-001 are essential reactants for the BMX-001-driven catalysis of protein thiols oxidation, resulting in tumor suppression [6, 7]. Numerous studies, as well as the most recent study on ovarian cancer cells OV90 and OVCD, corroborated high tumor H_2_O_2_ levels [6, 7, 9, 33]. Further on, up to 10-fold higher Mn porphyrin levels were found in 4T1 mouse breast tumor vs. normal tissue, depending upon the type of Mn porphyrin [6, 13]. In this study, the levels of BMX-001 in tumor were significantly higher than in normal muscle tissue from nontumor-bearing leg (Figure 7). Statistical analysis of the BMX-001 levels in tumor and nontumor tissues is detailed in Table S2. No impact of Asc was seen on BMX-001 levels in tumors and muscles, in agreement with data reported on analogous Mn porphyrin, MnTE-2-PyP^5+^, in a 4T1 breast cancer mouse flank study [6, 13]. As already indicated in Section 1, the higher the levels of BMX-001 and the higher the oxidative stress, the higher is the oxidative damage to tumor proteins. Consequently, higher is the suppression of tumor growth [6, 7].

## 4. Discussion

The redox-active *ortho* isomeric Mn(III) *meso*-tetrakis(*N*-ethylpyridinium-2-yl) porphyrin, MnTE-2-PyP^5+^ (BMX-010), has been our first clinical candidate developed [6, 7]. With five positive charges, though, the molecule is hydrophilic. While it crossed cellular membranes and entered heart mitochondria, it lacks lipophilicity to reach brain to a significant extent [6, 7]. We have subsequently designed two other more lipophilic lead candidates. We firstly lengthened the ortho-ethylpyridyl chains from methyl to ethyl to hexyl to octyl. Among those, the hexyl analog, MnTnHex-2-PyP^5+^ (Mn[III] *meso*-tetrakis[*N*-n-hexylpyridinium-2-yl)]), was chosen as a second lead compound. Yet, due to its long lipophilic chains and polar/charged center, MnTnHex-2-PyP^5+^ exerted surfactant property and therefore toxicity [6, 7]. To optimize the balance between toxicity and lipophilicity, oxygen atoms were inserted into hexyl chains [6]. Such structural modification of MnTnHex-2-PyP^5+^ gave rise to a third-generation lead compound, butoxyethyl analog, MnTnBuOE-2-PyP^5+^ (BMX-001) [6]. BMX-001 has approximately 6900-fold higher lipophilicity than MnTE-2-PyP^5+^ but significantly lower toxicity than the similarly lipophilic MnTnHex-2-PyP^5+^ [6].

Redox-active metalloporphyrins were also utilized by Daniela Salvemini's group. She has studied the mechanism of peroxynitrite-induced inflammation in a rodent model of both pain and peripheral neuropathy [36, 37] and has collaborated with us on Mn porphyrins [11, 37, 38]. Along with Neumann's group, she has initially used Mn and Fe porphyrins, MnTE-2-PyP^5+^ and FeTM-4-PyP^5+^, that bear four cationic charges on the periphery, to study suppression of chemotherapy-induced neuropathy in a rodent model [11, 37]. She has assigned their efficacy in suppression of chemotherapy-induced neuropathy to their ability to scavenge peroxynitrite, whereby suppressing inflammatory pathways involving NF-кB and TNF-α [37]. Further, Neumann's and Salvemini's groups developed Mn(III) tetracyclohexenylporphyrin (TCHP) and bishydroxyphenyldipyrromethene analogs that bear no charges on the periphery, aiming at oral availability [39, 40]. Two of those compounds [40] showed analgesic effects in reducing PTX-induced neuropathy in a rodent model when given orally [11]; no report on further clinical development of those analogs is available.

**Scheme 4:**
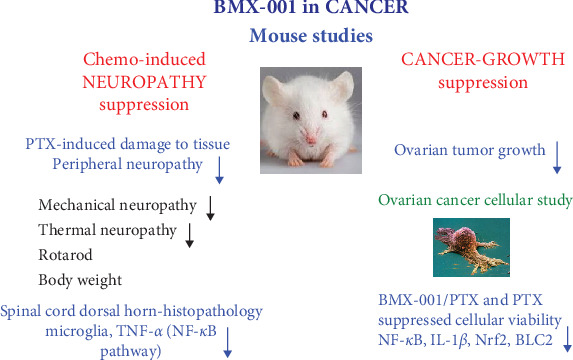
Mn porphyrin, MnTnBuOE-2-PyP^5+^ (BMX-001) suppressed PTX-induced neuropathy and CAOV2 high-grade serous ovarian tumor growth. BMX-001 suppressed PTX-induced peripheral neuropathy as measured by Von-Frey filaments and Hot plate, and microglia- and TNF-α-driven neuroinflammation in spinal cord dorsal horn. BMX-001 further suppressed tumor growth in a mouse ovarian sc xenograft tumor study. The expression of NF-кB, IL-1β, Nrf2, and BCL2, was reduced by PTX and BMX-001/PTX in ovarian cancer cellular study.

The wealth of data on the efficacy of BMX-001 in cellular and animal models and the satisfactory safety/toxicity profile demonstrated in Phase I glioma clinical trial, promoted its development into Phase II glioma [10] and multiple brain metastases trials. Such data motivated us to explore the efficacy of this compound in reducing PTX-induced peripheral neuropathy while suppressing ovarian tumor growth (Scheme 4).

The peripheral neuropathy in mouse paws was assessed by Von-Frey filaments (mechanical allodynia) and hot plate (thermal allodynia) (Figure 1). Also, the mouse's muscular coordination and general physical condition were assessed by their ability to walk on rotarod, while systemic toxicity was determined by measurement of body weight (Figure 1). The three trials were performed with four groups, *n* = 10 per group (vehicle, PTX, BMX-001, and PTX/BMX-001) and showed that BMX-001 was able to significantly reduce PTX-induced neuropathy when given either at 2 mg/kg/day, or at 6.25-fold lower and clinically relevant dosing of 0.8 mg/kg twice weekly (with 1.6 mg/kg loading dose). Similar effects at both dosing regimens corroborated the catalytic property of BMX-001 as it regenerates itself via cycling with biological molecules, such as Asc and protein thiols [6, 7].

The suppression of PTX-induced neuropathy by BMX-001 occurs via inhibition of neuroinflammation in the dorsal horn of spinal cord due to reduced number of microglia and levels of TNF-α (Figure 2). TNF-α has been central in orchestrating inflammatory and immune responses, with NF-кB and mitogen-activated kinases (MAPK) involved [32, 38, 41–43]. Mn porphyrin-driven protein oxidation and subsequent modification of the activity of numerous proteins such as NF-кB [6, 7, 13], MAPKs, and Keap1 factor of Keap1-Nrf2 signaling pathway was reported [7, 13, 44]. Oxidation of Keap1 by Mn porphyrin in normal cells/tissues activates Nrf2, which leads to the upregulation of prosurvival proteins (endogenous antioxidative defenses) [9, 44]. Both the inhibition of NF-кB activity and the activation of Nrf2 by BMX-001 likely account for suppression of PTX-induced neuropathy. The ability of BMX-001 to suppress chemotherapy-induced neuropathy may be of wider significance and could be applied to any other cancer where PTX, or other chemotherapeutics that induce neuropathy, are prescribed. Several classical chemotherapeutics such as platinum, vinca alkaloids, and taxanes are known to induce neuropathy in cancers such as lung cancer, lymphoma, myeloma, and in tumors pressing nerves [3–5, 45]

In addition to suppressing PTX-induced peripheral neuropathy, BMX-001 also exhibited strong anticancer effect in subcutaneous xenograft mouse model of aggressive high-grade serous CAOV2 ovarian cancer line (Scheme 4). The effect of BMX-001 was of a similar magnitude to that of PTX (Figure 6). The robust effect of BMX-001, as a single drug, was presumably due to a high oxidative stress in chemoresistant aggressive CAOV2 cell line and high BMX-001 tumor levels (Figure 7) which in turn supported BMX-001/H_2_O_2_-driven catalysis of proteins oxidation whereby promoting tumor growth suppression (Sections 1 and 3 for details). In cancers thus far explored [7], significant in vivo anticancer effect was demonstrated only when Mn porphyrin was combined with additional source/s of H_2_O_2_, such as radiation, chemotherapy, and exogenous Asc.

The mechanism of anticancer effect was addressed in CAOV2 ovarian cancer cellular studies. At 72 h of cancer growth, PTX and PTX/BMX-001 significantly reduced cell viability (Figure 3). Further, PTX and to a lesser extent PTX/BMX-001 reduced the expression of prosurvival NF-кB, IL-1β, Nrf2, and BCL2 proteins (Figure 4). Based on our data on glioblastoma multiforme [6, 7], breast cancer [6, 7, 13], lymphoma [6], and Chaiswing et al. data on ovarian cancer [9] where BMX-001 or its analogs were administered along with radiation-, chemotherapy-, or Asc-based sources of H_2_O_2_, the proteins affected by BMX-01/PTX were likely oxidized. The oxidation of proteins has been reportedly accompanied by the modification of their activities (Section 3) [6, 7, 13].

The impact of BMX-001/PTX on BCL2 is noteworthy as this antiapoptotic protein (controlled by NF-кB pathway [35]) is reportedly upregulated in ovarian cancer [34, 35]. Importantly, many anticancer drugs activate Nrf2 pathway, consequently inducing chemoresistance [46]. Namely, the activation of Nrf2 would have otherwise upregulated endogenous antioxidants, which would help tumor fight high H_2_O_2_ levels, thereby, promoting its survival. That seems not to be the case when BMX-001 was combined with PTX in our cellular study and with CB in the study of Chaiswing's team on ovarian cancer cell lines. The BMX-001/CB-driven suppression of Nrf2 activity in aggressive ovarian OV90 and OVCD cell lines was discussed with regard to decreased Trx1-Nrf2 interaction while increased Trx1-Keap 1 interaction [9]. Trx1-Keap 1 interaction promoted Nrf2 ubiquitination and decreased Nrf2 translocation to the nucleus.

Ours is the first in vivo study that demonstrated the ability of redox-active Mn porphyrin, BMX-001, to suppress peripheral neuropathy induced by PTX, a drug commonly used in the treatment of ovarian cancer. It is also the first *in vivo* study where BMX-001, as a single drug, suppressed tumor growth of an aggressive ovarian cancer cell line, and to a similar extent as that of PTX. Such a strong anticancer efficacy of BMX-001 could allow for the lower dosing of PTX, reducing in turn its toxicity to normal tissue. Our data add to the spectrum of differential effects of redox-active Mn porphyrins [6, 7]. We have recently submitted the SBIR proposal to support the clinical development of BMX-001 in ovarian and endometrial cancers.

## 5. Conclusions

We have herein provided evidence that redox-active Mn porphyrin, BMX-001 (MnTnBuOE-2-PyP^5+^), bears therapeutic potential for reducing chemotherapy-induced peripheral neuropathy at clinically relevant dosing via microglia/TNF-α signaling involved in suppression of neuroinflammation. Importantly, we have also demonstrated that, aside from the protective effect on neurons, BMX-001 is capable of suppressing tumor growth to a similar extent as PTX in a mouse high-grade serous CAOV2 ovarian cancer subcutaneous xenograft model. Thus, our data reveal strong therapeutic potential of BMX-001 for the treatment of gynecological cancers.

## Figures and Tables

**Table 1:** Statistical analysis of neuropathy testing on week 3 (end of treatment).

	Von Frey			Hot plate		
Groups compared	Trial 1	Trial 2	Trial 3	Trial 1	Trial 2	Trial 3
Vehicle vs. PTX	<0.0001	<0.0001	<0.0001	<0.0001	<0.0001	0.0007
Vehicle vs. BMX-001	<0.0001	<0.0001	0.0007	0.0312	0.8995	0.3747
Vehicle vs. PTX + BMX-001	0.0023	0.0011	<0.0001	0.0273	0.9820	0.2657
PTX vs. BMX-001	<0.0001	<0.0001	<0.0001	0.0087	<0.0001	0.0796
PTX vs. PTX + BMX-001	<0.0001	<0.0001	0.0031	0.0101	<0.0001	0.1303
BMX-001 vs. PTX + BMX-001	0.6822	0.2007	0.6502	>0.9999	0.7096	0.9963

*Note:* Two-way ANOVA with Tukey's multiple comparisons was performed for analysis of all neuropathy testing data and *p* values on week 3 for Von-Frey and hot plate tests are given. The difference in mean values of the groups compared is considered statistically significant if *p*  < 0.05. The most relevant result the statistically significant difference between neuropathy observed in PTX-only versus PTX/BMX-001 groups. Full statistical report, including rotarod and body weight data, is given in Table S1.

## Data Availability

All raw data related to the rotarod and body weight analyses of three trials of the neuropathy study (Figure S1), with the statistical analysis of all neuropathy data (Table S1), and the statistics of the BMX-001 levels in tumors and nontumors tissues (Table S2) will be available from the corresponding authors upon reasonable request.

## Supporting Information

Supporting Information Supporting Information consists of Figure S1 with plots of rotarod and body weight results; Table S1 with (1) summary of ANOVA results for neuro score tests, (2) variance of homogeneity, and (3) normality of distribution of PTX group vs. PTX/BMX-001 group data (most relevant in this work), followed by complete statistical analysis (ANOVA) of Von-Frey, hot plate, rotarod, and body weight data from neuropathy testing in Trials 1, 2, and 3; and Table S2 with statistics of BMX-001 levels in tumor and muscle from mouse cancer study showed in Figure 7.
