# Supplementary material for: Suppression of Paclitaxel-Induced Neuropathy and Ovarian Tumor Growth by Mn Porphyrin, MnTnBuOE-2-PyP5+ (BMX-001)
Source: Oxid Med Cell Longev. 2025 Aug 24;2025:6333148. doi: 10.1155/omcl/6333148 (PMC12399357; doi:10.1155/omcl/6333148)

**Supporting Information**

**Suppression of paclitaxel-induced neuropathy and ovarian tumor growth by Mn porphyrin, MnTnBuOE-2-PyP^5+^ (BMX-001)**

*^1^Ivan Spasojevic, ^2^Zhiqing Huang, ^1^Welida Tamires Alves da Silva, ^3#^Weina Duan, ^3#^Li Du, ^1&^Kathleen Chen, ^1^Jie Cao, ^3^Shasha Zhang, ^2^Hannah Lee, ^2^Gaomong Lo,* *^4^Artak Tovmasyan, ^5^Ines Batinic-Haberle ^3^Huaxin Sheng, and ^6^Angeles Alvarez Secord*

*^1^Department of Medicine, and PK/PD Core Laboratory, Duke Cancer Institute, Duke University School of Medicine, Durham, NC 27710, USA, ^2^Division of Reproductive Sciences, Department of Obstetrics and Gynecology, Duke Cancer Institute, Duke University School of Medicine, Durham, NC 27710, USA, ^3^Department of Anesthesiology, Multidisciplinary Neuroprotection Laboratories, Center for Perioperative Organ Protection, Duke University School of Medicine, Durham, NC 27710, USA, ^4^Department of Translational Neuroscience, Barrow Neurological Institute, Phoenix, AZ 85013, USA, ^5^Department of Radiation Oncology, Duke University School of Medicine, Durham, NC 27710, USA, ^6^Division of Gynecologic Oncology, Department of Obstetrics and Gynecology, Duke Cancer Institute, Duke University School of Medicine, Durham, NC 27710, USA*

**Peripheral Neuropathy**

***Rotarod and Body Weights***


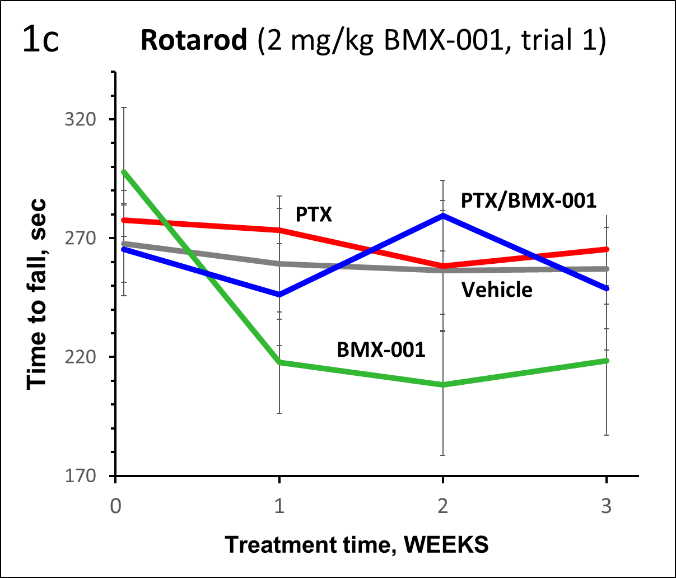

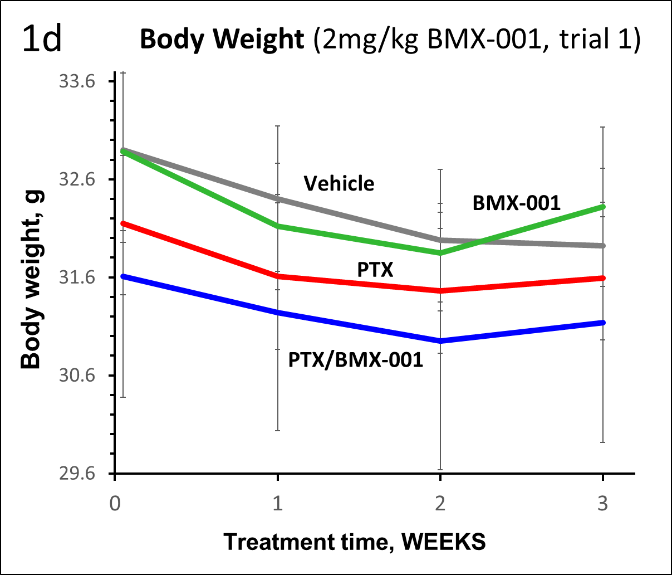


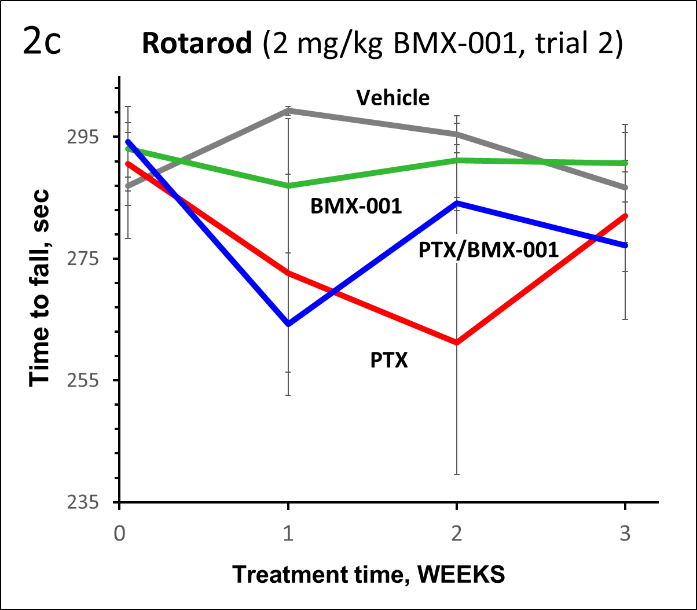

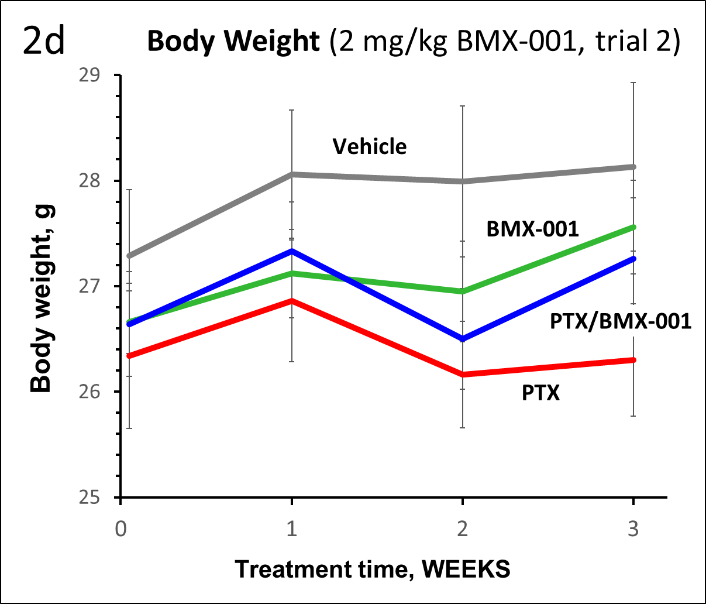


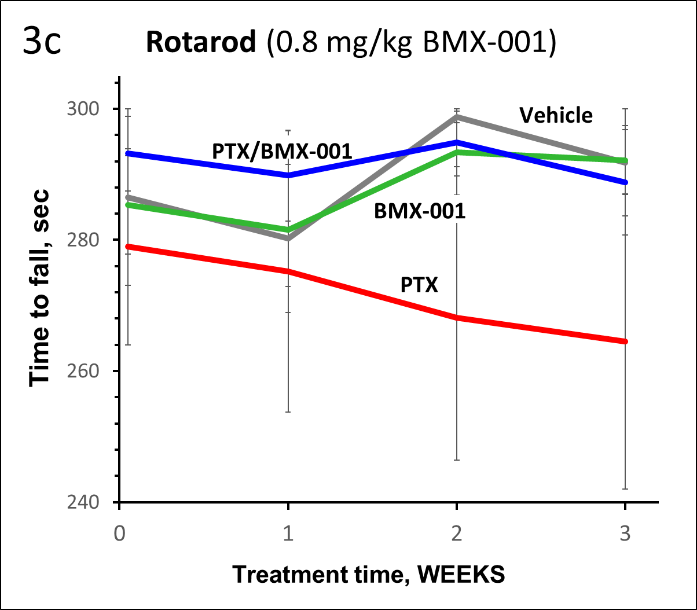

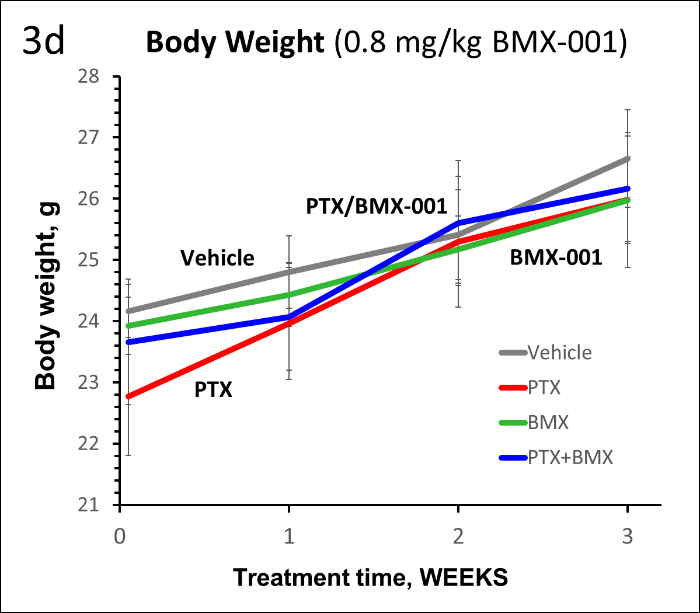


**Figure S1.** **The efficacy of BMX-001 in reducing PTX-induced neuropathy – rotarod and body weight.** The BMX-001 was given SC at 2 mg/kg/day in trials 1c/1d and 2c/2d. In a trial 3c/3b, the loading dose of 1.6 mg/kg of BMX-001 was given on a first Monday and was followed by twice weekly SC dosing of 0.8 mg/kg given on Fridays and Mondays. In all trials, the PTX was given IP at 10 mg/kg given every second day for four days. The systemic toxicity was assessed by body weight, while muscular coordination and general physical condition was evaluated by rotarod. All measurements were made a week before experiment (Week -1, “training”), before the treatments (Week 0), then once a week for 3 weeks. There was no significant difference in rotarod performance and body weight between experimental groups over the entire experiments, attesting to absence of systemic toxicity which would have otherwise introduced bias in the peripheral neuropathy assessment. Two-way ANOVA was performed on GraphPad Prism v.10. Tukey’s multiple comparisons tests were performed to determine differences in multi-groups experiments; for a full report please refer to Table 1S below. All data plotted are presented as mean ± SEM.

**Tables S1**. **Statistical analysis**. Two-way ANOVA and Tukey’s multiple comparisons were performed for analysis of data between the 4 study groups on weeks 0-3, using GraphPad Prism v.10 software package. *P < 0.05; **P < 0.01; ***P < 0.001; ****p<0.0001

**Summary of ANOVA results for neuro score tests**

**PTX group vs PTX+BMX001 group**

**COMPLETE STATISTICAL ANALYSIS (ANOVA) of Neuro Scores**

**Trial 1_Von-Frey (2 mg/kg BMX-001)**

**Trial 1_Hot Plate (2 mg/kg BMX-001)**

**Trial 1_Rotarod (2 mg/kg BMX-001)**

**Trial 1_Body Weight (2mg/kg BMX-001)**

**Trial 2_Von-Frey (2 mg/kg BMX-001)**

**Trial 2_Hot Plate (2 mg/kg BMX-001)**

**Trial 2_Rotarod (2 mg/kg BMX-001)**

**Trial 2_Body Weight (2 mg/kg BMX-001, trial 2)**

**Trial 3_Von-Frey (0.8 mg/kg BMX-001)**

**Trial 3_Hot Plate (0.8 mg/kg BMX-001)**

**Tral 3_Rotarod (0.8 mg/kg BMX-001)**

**Trial 3_Body Weight (0.8 mg/kg BMX-001)**

**Table S2. Statistics for the study: BMX-001 levels in tumor and muscle**


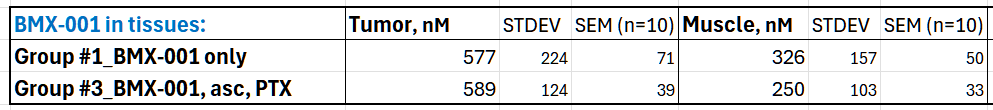


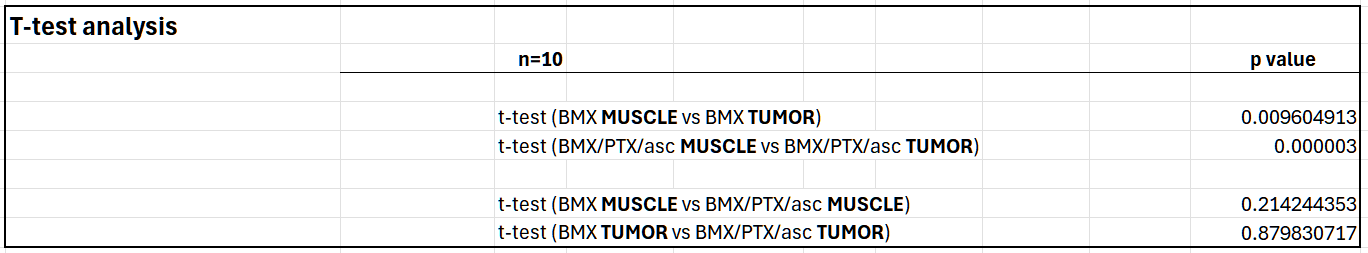

Supplement: Supporting Information — consists of Figure S1 with plots of rotarod and body weight results; Table S1 with (1) summary of ANOVA results for neuro score tests, (2) variance of homogeneity, and (3) normality of distribution of PTX group vs. PTX/BMX-001 group data (most relevant in this work), followed by complete statistical analysis (ANOVA) of Von-Frey, hot plate, rotarod, and body weight data from neuropathy testing in Trials 1, 2, and 3; and Table S2 with statistics of BMX-001 levels in tumor and muscle from mouse cancer study showed in Figure 7. [file 6333148.f1.docx]
